# The Effect of Vegan Protein-Based Diets on Metabolic Parameters, Expressions of Adiponectin and Its Receptors in Wistar Rats

**DOI:** 10.3390/nu8100643

**Published:** 2016-10-18

**Authors:** Jie-Hua Chen, Jia Song, Yan Chen, Qiang Ding, Anfang Peng, Limei Mao

**Affiliations:** 1 Department of Nutrition and Food Hygiene, Guangdong Provincial Key Laboratory of Tropical Disease Research, School of Public Health, Southern Medical University, Guangzhou 510515, Guangdong, China; siyanpijiehua@gmail.com (J.-H.C.); Jia_Song1991@126.com (J.S.); 2 Department of Nutrition and Food Hygiene, School of Public Health, Tongji Medical College, Huazhong University of Science and Technology, Wuhan 430030, Hubei, China; yan_chennutr@126.com (Y.C.); dq0306@163.com (Q.D.); nutritionpeng@163.com (A.P.)

**Keywords:** vegan protein, dietary intervention, metabolic syndromes, adiponectin, rats

## Abstract

Vegan protein-based diet has attracted increasing interest in the prevention of metabolic syndrome (MetS). Meanwhile, adiponectin has become a highly potential molecular target in the prevention of MetS. Our study will identify a potential vegan protein diet for the prevention of MetS using rat models. Thirty-six Wistar rats were randomly assigned into three groups and given diets containing one of the following proteins for 12 weeks: casein (CAS, control diet), soy protein (SOY), and gluten-soy mixed protein (GSM). Changes in metabolic parameters as well as the expressions of adiponectin and its receptors were identified. Compared to CAS diet, both SOY and GSM diets led to decreases in blood total cholesterol and triglycerides, but only GSM diet led to an increase in HDL-cholesterol; no marked difference was observed in blood glucose in all three groups; HOMA-IR was found lower only in SOY group. Among groups, the order of serum adiponectin level was found as GSM > SOY > CAS. Similar order pattern was also observed in expression of adiponectin in adipose tissue and AdipoR1 mRNA in skeletal muscle. Our results suggested for the first time that, besides SOY diet, GSM diet could also be a possible substitute of animal protein to prevent MetS.

## 1. Introduction

Metabolic syndrome (MetS) is a clustering of factors that increase the risk of developing obesity, hypertension, diabetes, and cardiovascular disease. Shifted paradigms of diet and lifestyle in modern times have led to a rapid rise of the prevalence of MetS, afflicting many people worldwide.

Adiponectin has been recognized as an independent protective factor for MetS owing to its regulatory effect in glucose and lipid metabolism, insulin sensitivity, and inflammatory response [1]. As an adipokine secreted mainly by adipose tissues, adiponectin has been shown to exert its action through its receptors AdipoR1, AdipoR2, and T-cadherin [2]. AdipoR1 and AdipoR2 are the major functional receptors. AdipoR1 is ubiquitously expressed with the greatest expression found in skeletal muscle, whereas AdipoR2 is mainly expressed in the liver [3,4]. Skeletal muscle and liver are primary sites in the body that are involved in glucose metabolism [5]. Yamauchi et al. (2003) found that the suppression of AdipoR expression led to the inhibition of glucose uptake in vitro [4]. It has been reported that the actions of adiponectin promoting glucose utilization and mitochondrial substrate oxidation in skeletal muscle are mediated through binding to receptors, especially AdipoR1 [6].

Different types of dietary proteins might interfere with the metabolic system. Recent studies showed that casein elevated blood cholesterol level [7], whereas the hypolipidemic effects were observed when casein was replaced by soy or other vegan proteins in rodent diets with all other nutrients kept relatively constant [8,9]. In addition, the favorable effects of soy protein on insulin response and adiponectin have been reported in monkeys [10]. No previous study however, has investigated longitudinally over a long period of time whether adiponectin and its receptors can be regulated by vegan proteins.

Cereal protein is the main protein in Chinese diets. Unlike casein and soy protein, cereal protein is not a complete protein, and it requires to be complemented with soy protein-rich food or other complete proteins to increase bioavailability. Definite effects of the mixture of soy and cereal protein on metabolism are yet to be elucidated. In the present study, two different types of vegan protein-based diets—soy diet and gluten-soy mixture diet—were employed in Wistar rats against the control diet (casein diet) to investigate the changes in metabolic parameters and expressions of adiponectin and its receptors in tissues, in an effort to identify a better protein-based diet to prevent MetS and its related chronic diseases.

## 2. Materials and Methods 

### 2.1. Animals and Diets

Thirty-six adult male Wistar rats (six weeks old, body weight 170–190 g) were provided by a local supplier for laboratory animals (Laboratory Animal Center of Hubei Province; Wuhan, China). Rats were housed individually and maintained at 22 °C ± 2 °C, 50% ± 10% humidity under 12–12 h light/dark cycle. All experimental protocols were reviewed and approved by Tongji Medical College Council of Animal Care Committee SCXK(Hubei)2003-2005. After acclimatization for a period of 1 week, rats were randomly assigned into three different groups and given diets containing 20% of one of the following proteins for 12 weeks: casein (CAS, *n* = 12), soy protein (SOY, *n* = 12) and wheat gluten-soy mixed protein (GSM, mixing ratio 1:1, *n* = 12). The 50:50 mixture of soy and wheat protein may be expected to produce an amino acid composition of high biological value based on amino acid content of gluten and soy protein [11,12]. Rat chow diets were prepared based on the Association of Official Analytical Chemists (AOAC) and AIN-93G formulas to meet the nutrient and energy requirements for the growth and development of rats [13]. Composition of rat chow diet of all groups is shown in Table 1. All rats had ad libitum access to drinking water.

The amount of feed consumed and the amount of excreted feces and urine by each rat were recorded daily. Body weights of rats were monitored weekly. Blood samples were collected after rats were fasted for 10 h overnight at the end of the study. Rats were then sacrificed by decapitation. Blood samples were centrifuged promptly after collection to obtain serum samples that were later stored at −80 °C. The peri-epididymal and perirenal adipose tissues, skeletal muscle tissue, and hepatic tissue were dissected, weighed, and frozen immediately in liquid nitrogen prior to storage at −80 °C.

### 2.2. Biochemical Measurement

Commercial ELISA kit (R & D Systems, Minneapolis, MN, USA) was used to measure serum adiponectin at the baseline and at the end of the study. Fasting blood glucose level, fasting serum insulin, serum total cholesterol, fasting triglyceride, and serum high density lipoprotein cholesterol (HDL-C) were determined by enzymatic colorimetric analysis using commercialized kits with details listed in Chen et al. (2015) [14].

### 2.3. Semi-Quantitative Reverse Transcriptase Polymerase Chain Reaction

Total RNA extraction of the epididymal adipose tissue, skeletal muscle tissue, and liver tissue were performed using Trizol reagent (Life Technologies, Carlsbad, CA, USA) for subsequent analysis of mRNA expression of adiponectin, AdipoR1 and AdipoR2. The concentration of RNA was determined using nucleic acid analyser (Eppendorf, Hamburg, Germany). For reverse transcriptase polymerase chain reaction, complementary DNA was synthesized from total RNA (3 μg) and later amplified using polymerase chain reaction (PCR). Specific primers used for PCR is shown in Table 2 β-actin served as a loading control.

Each PCR reaction contained the following reagents: 0.2 mmol/L dNTP (deoxyribonucleotide triphosphate), 1.5 μL complementary DNA, 0.25 μmol/L of each primer, 1 × PCR buffer, and 0.8 μL Taq polymerase. The PCR samples were incubated with initial 5 min denaturation followed by 33 cycles. Each cycle consisted of 94 °C for 30 s, 54 °C for 40 s, and 72 °C for 45 s for β-actin; or 94 °C for 30 s, 58 °C for 40 s, and 72 °C for 45 s for adiponectin; or 94 °C for 30 s, 57 °C for 40 s, and 72 °C for 45 s for AdipoR1/2; and 72 °C for 7 min at the final extension step in a PCR Thermocycler (Biometra, Gottingen, Germany).

The amplified DNA was then subjected to electrophoresis in 1.5% agarose gels for analysis in a gel imaging and analysis system (Biometra, Gottingen, Germany). The expression levels of adiponectin mRNA and AdipoR1/2 mRNA were shown using the absorbance ratio of target mRNA and β-actin mRNA.

### 2.4. Western Blot

Proteins of epididymal adipose tissue were extracted using RIPA lysis buffer containing phenylmethanesulfonyl fluoride (PMSF) and phosphatase inhibitor cocktails (all from KeyGEN BitoTECH, Nanjing, China), and quantified by bicinchonininc acid (BCA) protein assay kits (ExCell, Shanghai, China). Approximately 40 μg of denatured proteins were loaded and separated by SDS-PAGE (12% acrylamide), and then transferred to the polyvinylidene difluoride (PVDF) membranes (0.45 μm, Millipore, Bedford, MA, USA) using a wet-transfer system at 100 V for 60 min. After blocking with 5% non-fat milk, membranes were incubated with adiponectin specific (1:500 dilution) and β-actin specific primary antibodies (1:3000, all from Santa Cruz, Santa Cruz, CA, USA) overnight at 4 °C. Membranes were thereafter rinsed five times with Tris-buffered saline-Tween (TBST, 0.1% Tween) washing solution, followed by incubated with corresponding secondary antibodies (1:2000 dilution for anti-mouse IgG and 1:5000 dilution for anti-rabbit IgG, all from Santa Cruz, Santa Cruz, CA, USA) for 2 h at room temperature. After washing, strips in membranes were visualized using chemiluminescent peroxidase substrate (Millipore, Bedford, MA, USA) and Tanon-5200 chemical luminescence developing system (Tanon, Shanghai, China). β-actin served as the internal reference. The expressions of adiponectin and β-actin proteins in each group were determined by grey value analysis using Image J software (Bethesda, MD, USA).

### 2.5. Statistical Analysis

Data were expressed as means ± SEM. The results were statistically analysed by one-way ANOVA followed by Least Significant Difference (LSD)-test with SPSS version 12 software (SPSS Inc., Chicago, IL, USA). Transformation was applied to correct for unequal variances. In all tests, a *p* value less than 0.05 was considered statistically significant.

## 3. Results

### 3.1. Feed Intake and Body Weight

**Figure 1:**
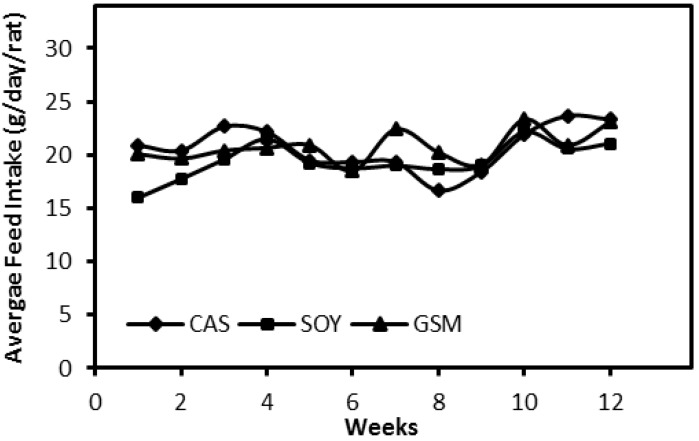
Weekly average feed intakes of three groups over the 12-week feeding period. Average feed intake of rats across all groups at the end of the study was statistically analyzed by one-way ANOVA followed by LSD-test and no significant difference was shown.

**Figure 2:**
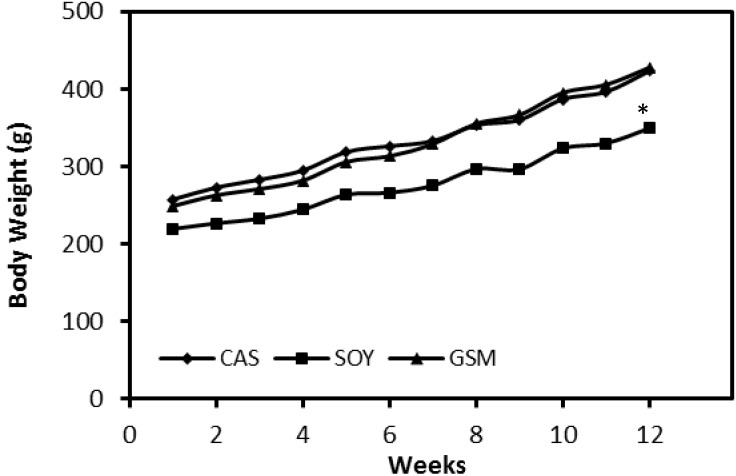
Change of body weights of rats from three groups over the 12-week feeding period. Body weights of rats at the end of the study were statistically analyzed by one-way ANOVA followed by LSD-test; * *p* < 0.05 versus CAS and GSM group.

The amount of feed intakes of all three groups demonstrated no difference over the 12-week feeding period (Figure 1). Growth rates of rats in SOY group were much slower than those in other two groups (Figure 2). Significant differences of body weights of rats between SOY and other two groups were detected in Week 4 (*p* < 0.05) and the differences were greater towards the end of the study (*p* < 0.001).

### 3.2. Changes in Metabolic Parameters

At the beginning, concentrations of serum lipid profiles demonstrated no differences among groups. At the end of the study, both SOY and GSM diets exhibited a greater decrease in triglyceride and total cholesterol as compared to control diet (CAS) (*p* < 0.05; Table 3). Though a higher level of HDL-C was observed only in the GSM group (*p* < 0.05), changes of HDL-C levels from 0 weeks to 12 weeks in both SOY and GSM groups were found to statistically differ from that in the CAS group (*p* > 0.05; Table 3).

No significant difference was detected in blood glucose among all groups at 0 weeks and 12 weeks (Table 3). Insulin levels decreased significantly only in SOY group as compared to CAS group (*p* < 0.05). Insulin resistance, as presented as HOMA-IR, was found significantly lower compared not only to CAS group, but to GSM group (*p* < 0.05). Nevertheless, changes in serum insulin levels and HOMA-IR from 0 weeks to 12 weeks were not different among all groups (*p* > 0.05).

**Figure 3:**
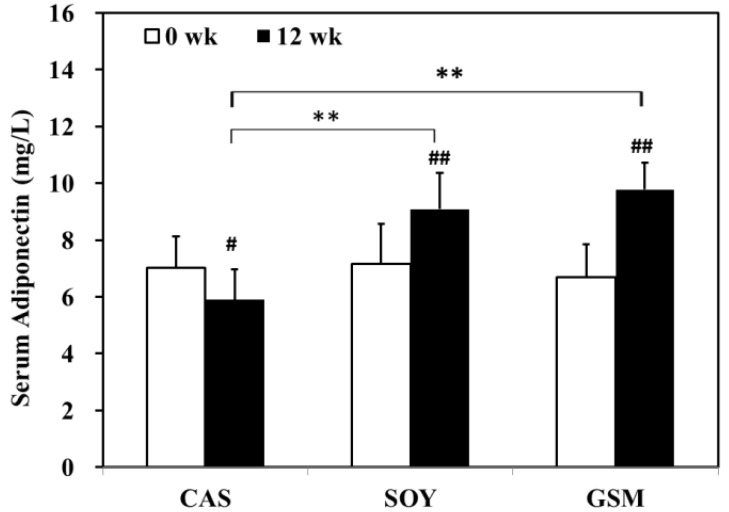
The effect of different dietary proteins on serum adiponectin. Serum adiponectin showed no significant difference among all three groups at the beginning of the study. During the 12-week period, serum adiponectin in the CAS group decreased significantly whereas that in SOY and GSM group increased significantly as compared to their respective baseline levels. Among groups, SOY and GSM groups demonstrated higher levels of serum adiponectin than that of CAS group at the end of the study. One-way ANOVA followed by an LSD-test was used to detect significant differences of the means, ** *p* < 0.001 versus CAS group; ^#^ *p* < 0.05 versus baseline; ^##^ *p* < 0.001 versus baseline.

All groups demonstrated a similar level of serum adiponectin at the beginning of the study (Figure 3). At the end of the study, serum adiponectin in CAS group decreased significantly whereas that in SOY and GSM group increased significantly as compared to their respective baseline levels. Among groups, SOY and GSM groups demonstrated higher levels of serum adiponectin than that of CAS group at the end of the study.

Different types of protein regimes also led to different visceral adipose tissue (VAT) accumulations (Table 4). At the end of the 12-week study, a significant decrease in mass of VAT was observed in SOY group as compared to the other two groups (*p* < 0.05). Percentages of wet weights of VAT to total body weights in SOY and GSM groups were significantly lower than that in CAS group (*p* < 0.05).

### 3.3. Gene Expression

**Figure 4:**
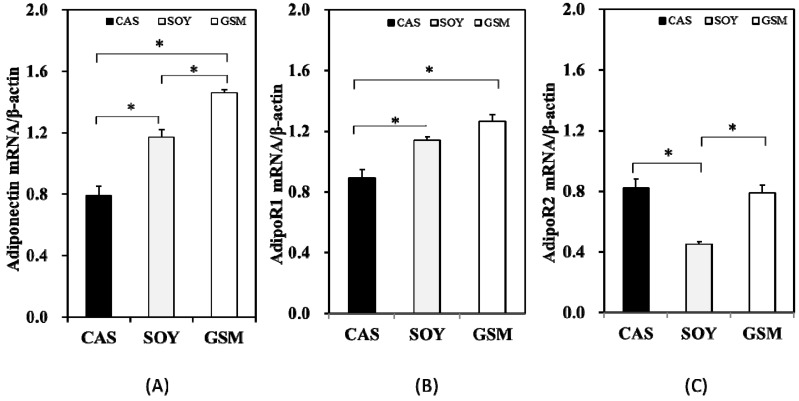
The effect of different dietary proteins on the expressions of adiponectin in adipose tissue, AdipoR1 in skeletal muscle and AdipoR2 in liver tissue. (**A**–**C**) demonstrate the expression levels (absorbance) of mRNA of adiponectin—AdipoR1 and AdipoR2—that were normalized against those of β-actin (served as a loading control). One-way ANOVA followed by LSD-test was used to detect significant differences of the means, * *p* < 0.05.

Results obtained from RT-PCR indicated that the order of expression levels of adiponectin mRNA in three groups was CAS < SOY < GSM (*p* < 0.05; Figure 4A). AdipoR1 mRNA expression levels in SOY and GSM groups were significantly higher than that in CAS group (Figure 4B) whereas expression level of AdipoR2 mRNA demonstrated a decrease as compared to the other two groups (Figure 4C).

### 3.4. Adiponectin Protein Expression

**Figure 5:**
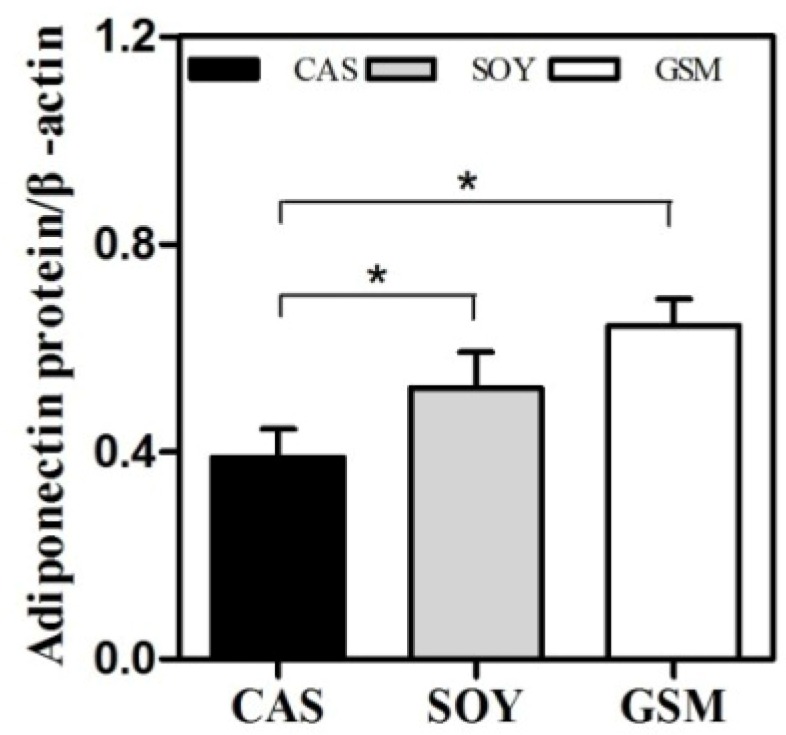
The effect of different dietary proteins on the expression of adiponectin protein in epididymal adipose tissue. Protein levels of adiponectin were normalized against that of β-actin (served as a loading control). One-way ANOVA followed by LSD-test was used to detect significant differences of the means, * *p* < 0.05.

Adiponectin protein levels in adipose tissues in SOY and GSM group were significantly higher than that in CAS group at the end of the study (*p* < 0.05; Figure 5). This finding was similar to the results of the expression of adiponectin mRNA in adipose tissue (Figure 4A) except that no significant difference was observed in adiponectin levels between SOY group and GSM group.

## 4. Discussion

MetS represents a collection of conditions that involve hypertension, glucose intolerance, insulin resistance, and dyslipidemia. Vegan protein-based diet has attracted increasing interest in the prevention and treatment of MetS [8,12,17]. In the present study, two different types of vegan protein-based diets—soy diet and gluten-soy mixture diet—were employed in Wistar rats against the control diet (casein diet) to investigate the changes in metabolic parameters and expressions of adiponectin and its receptors in tissues.

It is reported that, as compared to animal protein, vegan proteins enhance the activity of glucagon, stimulating fatty acid oxidation [9,17]. The anti-obesity effect of SOY compared with CAS reported in previous studies [18,19,20] was confirmed in the present study. As compared to the other two groups, body weight gain was significantly slower and visceral fat mass was markedly decreased in SOY group. GSM group, on the other hand, demonstrated no difference in body weight gain and visceral fat mass as compared to CAS group, though a reduction was observed when expressed as visceral fat mass %. Various studies have shown that VAT is involved in the development of MetS whereas subcutaneous adipose tissue is not [21,22,23,24]. Moreover, perirenal and peri-epididymal adipose tissue in rats were demonstrated to be more representative of visceral fat mass due to challenging dissection of mesenteric and subcutaneous fat [16]. Hence, total weight of perirenal and peri-epididymal adipose tissue was chosen for evaluation of visceral fat mass in the present study (Table 4).

The hypolipidemic effects observed when vegan proteins substitute for casein in rodent diets in various recent studies were also shown in the present study [8,9,25]. Consistent with results obtained from a meta-analysis [9], SOY diet in the present study led to reductions in total cholesterol and triglycerides but no significant increase was observed in HDL-C. In contrast to our result, a lower HDL-C level was observed in the SOY group in relation to the CAS group in a study conducted by Akahoshi et al. (2004) [18]. The other vegan protein diet in our study, GSM-based diet, on the other hand, led to a significant increase in HDL-C and reductions in total cholesterol and triglycerides. Our results suggested that GSM-based diet could be a possible better diet than SOY-based diet in terms of hypolipidemic effects. The underlying mechanism of the hypolipidemic activity of vegan proteins could be explained by the increased glucagon activity to stimulate hepatic fatty acid oxidation, the upregulation of the LDL receptor and downregulation of HMG-CoA reductase [9]. It has been suggested that the essential amino acids predominate in animal protein have greater efficacy for insulin secretion, whereas the non-essential amino acids provided more abundantly by vegan proteins have a greater impact on glucagon [9,26].

Maintaining relatively low levels of fasting insulin and HOMA-IR is shown to be beneficial for the prevention and treatment of diabetes. In good agreement with previous studies [18], SOY group manifested a lower insulin level and insulin resistance (HOMA-IR) than the other two groups. However, considering the percentage changes of serum insulin and HOMA-IR in all groups were not significantly different, the effect of SOY based diet on the improvement of insulin sensitivity in the present study was deemed minimal. Adiponectin demonstrates anti-atherogenic and anti-diabetic effects [27]. A high level of adiponectin is associated with better insulin sensitivity and improves glucose and lipid metabolism [28,29]. In accordance with findings obtained by Nagasawa et al. (2003), serum and adipose expression levels of adiponectin in Wistar rats were significantly higher in SOY group as compared to the control group [30]. Similar results to SOY group were also obtained in GSM group. The higher level of serum adiponectin in the SOY group observed in our study was contradictory to the findings obtained by Akahoshi et al. (2004), in which serum adiponectin was lower in SOY group than that in control group [18]. This discrepancy may be attributed to different feeding periods, 4 weeks in Akahoshi’s study (2004) and 12 weeks in the present study.

It has been elucidated that adiponectin exerts its action mainly through its receptors AdipoR 1 and 2, and mediates glucose uptake and FFA oxidation [2,4,31,32]. In rodents, AdipoR1 is ubiquitously expressed with the greatest expression found in skeletal muscle, whereas AdipoR2 is mainly expressed in the liver [5]. AdipoR1 disruption in mice led to obesity and impaired insulin resistance [31] with elevated levels of tissue triglycerides, inflammation, and oxidative stress [32]. In the present study, expression of AdipoR1 mRNA in skeletal muscle is higher in SOY and GSM groups as compared to that in the control group (GSM being the highest) whereas expression of AdipoR2 mRNA in liver tissue is found lower in SOY group but no difference in GSM group as compared to the control group. Individual effects of adiponectin receptors 1 and 2 on metabolic actions and energy metabolism have been reported by several studies [31,32]. This effect could be due to (1) distinct affinities of adiponectin receptors to two different types of adiponectins. AdipoR1 has been shown to express higher affinity to globular adiponectin but low affinity to full-length adiponectin, while AdipoR2 seems to be an intermediate-affinity receptor for both types of adiponectins [4]; (2) different receptor signaling pathways. Receptor 1 reduced gluconeogenesis through AMP-activated proteins kinase (AMPK) signaling pathways, while Receptor 2 stimulated glucose uptake and improved inflammation and oxidative stress through peroxisome proliferator-activated receptor (PPAR)-α signaling pathway. The present study for the first time demonstrated the effect of SOY and GSM diets on mRNA expressions of adiponectin receptors, suggesting the crucial roles of adiponectin receptors in regulation of metabolic parameters and that they could be possible therapeutic targets for treatment of MetS.

## 5. Conclusions 

Our results suggested that both SOY and GSM diets have shown desirable effects on controlling lipid profiles and modulating expressions of adiponectin and AdipoR1, though only SOY diet demonstrated slight improvement in insulin response. In addition, the effects of SOY and GSM diets on mRNA expressions of adiponectin receptors have been investigated for the first time. Our results suggested that, besides the traditionally SOY based diet, GSM based diet could also be a possible substitute of animal protein to ameliorate and prevent MetS, though the underlying mechanism requires further elucidation.

## Figures and Tables

**Table 1:** Composition of rat chow diet (g/kg).

Components	CAS Group	SOY Group	GSM Group
Casein ^a^ (g)	200.0	0.0	0.0
Soy protein isolates ^b^ (g)	0.0	400.0	200.0
Wheat Gluten isolates ^c^ (g)	0.0	0.0	142.8
Vitamin mix ** (g)	10.0	10.0	10.0
Mineral mix ** (g)	35.0	35.0	35.0
Sucrose (g)	50.0	50.0	50.0
Corn starch (g)	585.0	385.0	442.2
Fiber (g)	50.0	50.0	50.0
Lipid (g)	70.0	70.0	70.0
Total amount (g)	1000.0	1000.0	1000.0

** The composition of vitamin and mineral mix can be referred to AIN-93G; ^a^ Casein was purchased from Bodi Chemical Co., Ltd., Tianjin, China; ^b^ Soy protein isolates were purchased Qitian Biotechnology Co., Ltd., Anyang, China. Composition of soy protein isolates: protein 50%, carbohydrates and water 20%; ^c^ Whey Gluten isolates were purchased from Lianhua Gourmet Powder Co., Ltd., Henan, China. Composition of gluten isolates: protein 70%, carbohydrates and water 30%.

**Table 2:** Primers used for PCR.

	Sense Primer	Antisense Primer
**Adiponectin**	5′-TCACTCAGCATTCAGCGTAG-3′	5′-CTGATACTGGTCGTAGGTGAAG-3′
**AdipoR1**	5′-ACTGGACTATTCAGGGATTGC-3′	5′-CCATAGAAGTGGACGAAAGC-3′
**AdipoR2**	5′-TTGTGTATTCTTCCTGTGCC-3′	5′-CAGCACACAGATGACAATCA-3′
**β-actin**	5′-CATCACTATCGGCAATGAGC-3′	5′-GACAGCACTGTGTTGGCATA-3′

**Table 3:** Fasting blood lipid, metabolic, and insulin responses in rats fed with different dietary proteins ^†,^*.

	mmol/L				Insulin (IU/mL)	HOMA-IR ^#^
	Triglyceride	Total Cholesterol	HDL-C	Glucose		
**0-week**						
CAS	0.95 ± 0.28	1.99 ± 0.05	0.56 ± 0.11	7.54 ± 0.96	7.69 ± 1.79	2.56 ± 0.61
SOY	1.00 ± 0.21	2.14 ± 0.13	0.54 ± 0.10	7.53 ± 0.66	8.06 ± 1.66	2.37 ± 0.70
GSM	0.90 ± 0.19	2.14 ± 0.20	0.52 ± 0.07	7.07 ± 0.74	7.58 ± 1.99	2.39 ± 0.68
**12-week**						
CAS	1.55 ± 0.23	2.20 ± 0.24	0.62 ± 0.05	4.55 ± 0.77	9.21 ± 1.57	1.83 ± 0.51
SOY	0.79 ± 0.33 ^a^	1.77 ± 0.38 ^a^	0.69 ± 0.08	4.37 ± 0.83	7.45 ± 1.92 ^a^	1.31 ± 0.42 ^a,c^
GSM	0.67 ± 0.24 ^a,b^	1.63 ± 0.49 ^a^	0.79 ± 0.06 ^a^	4.82 ± 0.65	8.23 ± 1.86	1.77 ± 0.57
**Change (%)** ^†^						
CAS	48.7 ± 20.2	13.2 ± 10.2	−9.5 ± 20.8	−40.7 ± 15.0	16.4 ± 24.1	−32.1 ± 19.5
SOY	−19.5 ± 11.9 ^a^	−15.0 ± 9.0 ^a^	19.5 ± 15.2 ^a^	−39.7 ± 15.7	−4.0 ± 23.6	−42.3 ± 21.5
GSM	−46.4 ± 16.9 ^a,b^	−19.1 ± 11.8 ^a,b^	45.1 ± 18.1 ^a,b^	−32.9 ± 11.2	16.1 ± 30.7	−21.9 ± 26.5

^†^ Abbreviations: CAS: casein; SOY: soy protein; GSM: gluten-soy mixed protein; HDL-C: high density lipoprotein cholesterol; * Blood samples were collected at the end of the study; Data was presented as arithmetic mean ±1 SD (*n* = 12 for each group); ^#^ HOMA-IR (Homeostasis Model Assessment of Insulin Resistance) = Fasting Blood Glucose (mmol/L) × Fasting Insulin (mIU/L)/22.5 [15]; ^†^ Change (%) = (12 week–0 weeks)/0 week × 100%; ^a^ *p* < 0.05 versus CAS group; ^b^ *p* < 0.05 versus SOY group; ^c^ *p* < 0.05 versus GSM group.

**Table 4:** Effect of different dietary proteins on visceral adipose tissue ^†^.

Group	Visceral Fat Mass (g) ^#,^ *	Visceral Fat Mass (%) *^, Δ^
CAS	11.57 ± 1.67	2.62 ± 0.33
SOY	7.15 ± 1.71 ^a, b^	1.94 ± 0.46 ^a^
GSM	9.95 ± 1.08	2.13 ± 0.21 ^a^

^†^ Abbreviations: CAS: casein; SOY: soy protein; GSM: gluten-soy mixed protein; * Data was presented as arithmetic mean ± 1 SD *n* = 12 for each group; ^#^ Visceral Fat Mass (g) = total perirenal adipose tissue (g) + total peri-epididymal adipose tissue (g) [16]; ^Δ^ Visceral fat mass (%) = Visceral fat mass/Body weight × 100; ^a^ *p* < 0.05 versus CAS group; ^b^ *p* < 0.05 versus GSM group.
